# Supplementary material for: Pyruvate Dehydrogenase Kinase 1 inhibition mediated oxidative phosphorylation enhancement in cartilage promotes osteoarthritis progression
Source: BMC Musculoskelet Disord. 2023 Jul 20;24:597. doi: 10.1186/s12891-023-06585-6 (PMC10357736; doi:10.1186/s12891-023-06585-6)
Supplement: Supplementary file 1 — Additional file 1. [file 12891_2023_6585_MOESM1_ESM.zip › KEGG.Gsea.1653622432464/KEGG_ARACHIDONIC_ACID_METABOLISM.html]

Details for gene set KEGG\_ARACHIDONIC\_ACID\_METABOLISM[GSEA]

|  || Dataset | 55457\_collapsed\_to\_symbols.cls.cls #OA\_versus\_normal.cls.cls #OA\_versus\_normal\_repos |
| Phenotype | cls.cls#OA\_versus\_normal\_repos |
| Upregulated in class | OA |
| GeneSet | KEGG\_ARACHIDONIC\_ACID\_METABOLISM |
| Enrichment Score (ES) | 0.2541638 |
| Normalized Enrichment Score (NES) | 1.0205644 |
| Nominal p-value | 0.40941176 |
| FDR q-value | 0.5649324 |
| FWER p-Value | 1.0 |
Table: GSEA Results Summary

  

Fig 1: Enrichment plot: KEGG\_ARACHIDONIC\_ACID\_METABOLISM      
 Profile of the Running ES Score & Positions of GeneSet Members on the Rank Ordered List

  

| SYMBOL | TITLE | RANK IN GENE LIST | RANK METRIC SCORE | RUNNING ES | CORE ENRICHMENT || 1 | LTA4H | leukotriene A4 hydrolase [Source:HGNC Symbol;Acc:HGNC:6710] | 18 | 1.114 | 0.0691 | Yes |
| 2 | PTGS1 | prostaglandin-endoperoxide synthase 1 [Source:HGNC Symbol;Acc:HGNC:9604] | 163 | 0.845 | 0.1113 | Yes |
| 3 | LTC4S | leukotriene C4 synthase [Source:HGNC Symbol;Acc:HGNC:6719] | 223 | 0.804 | 0.1576 | Yes |
| 4 | CBR1 | carbonyl reductase 1 [Source:HGNC Symbol;Acc:HGNC:1548] | 598 | 0.624 | 0.1680 | Yes |
| 5 | PLA2G12A | phospholipase A2 group XIIA [Source:HGNC Symbol;Acc:HGNC:18554] | 600 | 0.623 | 0.2073 | Yes |
| 6 | HPGDS | hematopoietic prostaglandin D synthase [Source:HGNC Symbol;Acc:HGNC:17890] | 605 | 0.622 | 0.2463 | Yes |
| 7 | CBR3 | carbonyl reductase 3 [Source:HGNC Symbol;Acc:HGNC:1549] | 936 | 0.531 | 0.2542 | Yes |
| 8 | EPHX2 | epoxide hydrolase 2 [Source:HGNC Symbol;Acc:HGNC:3402] | 1659 | 0.405 | 0.2236 | No |
| 9 | GPX7 | glutathione peroxidase 7 [Source:HGNC Symbol;Acc:HGNC:4559] | 1919 | 0.370 | 0.2269 | No |
| 10 | AKR1C3 | aldo-keto reductase family 1 member C3 [Source:HGNC Symbol;Acc:HGNC:386] | 1996 | 0.361 | 0.2438 | No |
| 11 | PTGIS | prostaglandin I2 synthase [Source:HGNC Symbol;Acc:HGNC:9603] | 2654 | 0.284 | 0.2107 | No |
| 12 | PLA2G4A | phospholipase A2 group IVA [Source:HGNC Symbol;Acc:HGNC:9035] | 2656 | 0.284 | 0.2286 | No |
| 13 | PLA2G10 | phospholipase A2 group X [Source:HGNC Symbol;Acc:HGNC:9029] | 3315 | 0.220 | 0.1913 | No |
| 14 | GPX5 | glutathione peroxidase 5 [Source:HGNC Symbol;Acc:HGNC:4557] | 3558 | 0.198 | 0.1849 | No |
| 15 | PLA2G3 | phospholipase A2 group III [Source:HGNC Symbol;Acc:HGNC:17934] | 3648 | 0.190 | 0.1901 | No |
| 16 | ALOX5 | arachidonate 5-lipoxygenase [Source:HGNC Symbol;Acc:HGNC:435] | 3776 | 0.179 | 0.1915 | No |
| 17 | TBXAS1 | thromboxane A synthase 1 [Source:HGNC Symbol;Acc:HGNC:11609] | 4338 | 0.125 | 0.1558 | No |
| 18 | PTGES | prostaglandin E synthase [Source:HGNC Symbol;Acc:HGNC:9599] | 4399 | 0.121 | 0.1587 | No |
| 19 | PLA2G2D | phospholipase A2 group IID [Source:HGNC Symbol;Acc:HGNC:9033] | 5056 | 0.069 | 0.1121 | No |
| 20 | GGT1 | gamma-glutamyltransferase 1 [Source:HGNC Symbol;Acc:HGNC:4250] | 5177 | 0.058 | 0.1064 | No |
| 21 | PTGDS | prostaglandin D2 synthase [Source:HGNC Symbol;Acc:HGNC:9592] | 5583 | 0.025 | 0.0765 | No |
| 22 | GPX2 | glutathione peroxidase 2 [Source:HGNC Symbol;Acc:HGNC:4554] | 5808 | 0.008 | 0.0596 | No |
| 23 | CYP2C19 | cytochrome P450 family 2 subfamily C member 19 [Source:HGNC Symbol;Acc:HGNC:2621] | 6089 | -0.011 | 0.0385 | No |
| 24 | CYP2C18 | cytochrome P450 family 2 subfamily C member 18 [Source:HGNC Symbol;Acc:HGNC:2620] | 6210 | -0.022 | 0.0305 | No |
| 25 | PTGES2 | prostaglandin E synthase 2 [Source:HGNC Symbol;Acc:HGNC:17822] | 6269 | -0.025 | 0.0276 | No |
| 26 | PLA2G5 | phospholipase A2 group V [Source:HGNC Symbol;Acc:HGNC:9038] | 6772 | -0.061 | -0.0076 | No |
| 27 | GPX4 | glutathione peroxidase 4 [Source:HGNC Symbol;Acc:HGNC:4556] | 7115 | -0.089 | -0.0286 | No |
| 28 | CYP2B6 | cytochrome P450 family 2 subfamily B member 6 [Source:HGNC Symbol;Acc:HGNC:2615] | 7445 | -0.114 | -0.0469 | No |
| 29 | PLA2G2E | phospholipase A2 group IIE [Source:HGNC Symbol;Acc:HGNC:13414] | 7486 | -0.117 | -0.0426 | No |
| 30 | PLA2G1B | phospholipase A2 group IB [Source:HGNC Symbol;Acc:HGNC:9030] | 8232 | -0.173 | -0.0896 | No |
| 31 | CYP2C9 | cytochrome P450 family 2 subfamily C member 9 [Source:HGNC Symbol;Acc:HGNC:2623] | 8595 | -0.201 | -0.1051 | No |
| 32 | CYP4A11 | cytochrome P450 family 4 subfamily A member 11 [Source:HGNC Symbol;Acc:HGNC:2642] | 8654 | -0.205 | -0.0967 | No |
| 33 | CYP4F3 | cytochrome P450 family 4 subfamily F member 3 [Source:HGNC Symbol;Acc:HGNC:2646] | 9224 | -0.250 | -0.1251 | No |
| 34 | GGT5 | gamma-glutamyltransferase 5 [Source:HGNC Symbol;Acc:HGNC:4260] | 9361 | -0.261 | -0.1192 | No |
| 35 | CYP4F2 | cytochrome P450 family 4 subfamily F member 2 [Source:HGNC Symbol;Acc:HGNC:2645] | 9599 | -0.283 | -0.1198 | No |
| 36 | ALOX12B | "arachidonate 12-lipoxygenase, 12R type [Source:HGNC Symbol;Acc:HGNC:430]" | 9617 | -0.284 | -0.1031 | No |
| 37 | CYP2J2 | cytochrome P450 family 2 subfamily J member 2 [Source:HGNC Symbol;Acc:HGNC:2634] | 9642 | -0.287 | -0.0868 | No |
| 38 | ALOX12 | "arachidonate 12-lipoxygenase, 12S type [Source:HGNC Symbol;Acc:HGNC:429]" | 9683 | -0.290 | -0.0716 | No |
| 39 | PLA2G2A | phospholipase A2 group IIA [Source:HGNC Symbol;Acc:HGNC:9031] | 10152 | -0.336 | -0.0867 | No |
| 40 | PLA2G2F | phospholipase A2 group IIF [Source:HGNC Symbol;Acc:HGNC:30040] | 10628 | -0.386 | -0.0993 | No |
| 41 | CYP2E1 | cytochrome P450 family 2 subfamily E member 1 [Source:HGNC Symbol;Acc:HGNC:2631] | 10988 | -0.428 | -0.1001 | No |
| 42 | PLA2G6 | phospholipase A2 group VI [Source:HGNC Symbol;Acc:HGNC:9039] | 11128 | -0.446 | -0.0827 | No |
| 43 | ALOX15B | arachidonate 15-lipoxygenase type B [Source:HGNC Symbol;Acc:HGNC:434] | 11394 | -0.486 | -0.0726 | No |
| 44 | ALOX15 | arachidonate 15-lipoxygenase [Source:HGNC Symbol;Acc:HGNC:433] | 11405 | -0.488 | -0.0425 | No |
| 45 | GPX3 | glutathione peroxidase 3 [Source:HGNC Symbol;Acc:HGNC:4555] | 11407 | -0.488 | -0.0117 | No |
| 46 | CYP2C8 | cytochrome P450 family 2 subfamily C member 8 [Source:HGNC Symbol;Acc:HGNC:2622] | 11958 | -0.585 | -0.0175 | No |
| 47 | JMJD7-PLA2G4B | JMJD7-PLA2G4B readthrough [Source:HGNC Symbol;Acc:HGNC:34449] | 12076 | -0.614 | 0.0122 | No |
| 48 | PTGS2 | prostaglandin-endoperoxide synthase 2 [Source:HGNC Symbol;Acc:HGNC:9605] | 12641 | -0.820 | 0.0202 | No |
Table: GSEA details [plain text format]

  

Fig 2: KEGG\_ARACHIDONIC\_ACID\_METABOLISM      
 Blue-Pink O' Gram in the Space of the Analyzed GeneSet

  

Fig 3: KEGG\_ARACHIDONIC\_ACID\_METABOLISM: Random ES distribution      
 Gene set null distribution of ES for **KEGG\_ARACHIDONIC\_ACID\_METABOLISM**
